# The associations of fat tissue and muscle mass indices with all-cause mortality in patients undergoing hemodialysis

**DOI:** 10.1371/journal.pone.0211988

**Published:** 2019-02-13

**Authors:** Takahiro Yajima, Maiko Arao, Kumiko Yajima, Hiroshi Takahashi, Keigo Yasuda

**Affiliations:** 1 Department of Nephrology, Matsunami General Hospital, Gifu, Japan; 2 Department of Internal Medicine, Matsunami General Hospital, Gifu, Japan; 3 Division of Medical Statistics, Fujita Health University School of Medicine, Aichi, Japan; International University of Health and Welfare, School of Medicine, JAPAN

## Abstract

Protein-energy wasting, which involves loss of fat and muscle mass, is prevalent and is associated with mortality in hemodialysis (HD) patients. We investigated the associations of fat tissue and muscle mass indices with all-cause mortality in HD patients. The study included 162 patients undergoing HD. The fat tissue index (FTI) and skeletal muscle mass index (SMI), which represent respective tissue masses normalized to height squared, were measured by bioimpedance analysis after dialysis. Patients were divided into the following four groups according to the medians of FTI and SMI values: group 1 (G1), lower FTI and lower SMI; G2, higher FTI and lower SMI; G3, lower FTI and higher SMI; and G4, higher FTI and higher SMI. The associations of the FTI, SMI, and body mass index (BMI) with all-cause mortality were evaluated. During a median follow-up of 2.5 years, 29 patients died. The 5-year survival rates were 48.6%, 76.1%, 95.7%, and 87.4% in the G1, G2, G3, and G4 groups, respectively (P = 0.0002). The adjusted hazard ratio values were 0.34 (95% confidence interval [CI] 0.10–0.95, P = 0.040) for G2 vs. G1, 0.13 (95%CI 0.01–0.69, P = 0.013) for G3 vs. G1, and 0.25 (95%CI 0.07–0.72, P = 0.0092) for G4 vs. G1, respectively. With regard to model discrimination, on adding both FTI and SMI to a model with established risk factors, the C-index increased significantly when compared with the value for a model with BMI (0.763 vs. 0.740, P = 0.016). Higher FTI and/or higher SMI values were independently associated with reduced risks of all-cause mortality in HD patients. Moreover, the combination of the FTI and SMI may more accurately predict all-cause mortality when compared with BMI. Therefore, these body composition indicators should be evaluated simultaneously in this population.

## Introduction

Protein-energy wasting (PEW), defined as the loss of body protein mass and fuel reserves, is a common complication of chronic kidney disease and is an important predictor of mortality in patients with end-stage renal disease undergoing hemodialysis (HD) [1, 2]. Many epidemiologic studies have reported that a high body mass index (BMI) is associated with better survival in this population, and this phenomenon is referred to as the “obesity paradox” [3, 4]. However, BMI does not discriminate between body fat mass and muscle mass [5, 6]. We have recently reported that abdominal fat levels measured by computed tomography were negatively associated with risks for all-cause mortality in HD patients [7]. As a limitation of the study, muscle mass, another component of PEW, could not be evaluated at the same time. On the other hand, bioelectrical impedance analysis (BIA), which is commonly used in the area of nephrology to evaluate not only dry weight but also nutritional status in HD patients, enables the estimation of body composition [8,9]. The fat tissue index (FTI) and skeletal muscle mass index (SMI), which represent respective tissue masses adjusted for height squared, have been used to evaluate fat mass and muscle mass, respectively, and the SMI is used for the diagnosis of sarcopenia when muscle mass is measured by BIA [10–12]. Different results have been reported with regard to the relationship between body composition and mortality [13–16], and few studies have evaluated the associations between both fat mass and muscle mass and all-cause mortality simultaneously [10,17]. Furthermore, it is unknown whether mortality can be more accurately predicted with body composition indicators, such as the FTI and SMI, than with BMI.

The present study aimed to investigate the associations of the FTI and SMI with all-cause mortality and determine whether mortality can be more accurately predicted with these body composition indicators than with BMI in patients undergoing HD.

## Materials and methods

### Patients and assessments

A total of 162 patients undergoing HD for more than 6 months, and who had undergone BIA as part of a monthly examination at the outpatient clinic of Matsunami General Hospital, were enrolled between April 2012 and March 2018, and were followed up. In this retrospective study, all patient data were fully anonymized before we accessed them and ethics committee waived the requirement for informed consent. This study adhered to the principles of the Declaration of Helsinki, and the study protocol was approved by the ethics committee of Matsunami General Hospital (No. 381).

The following patient data were collected from medical records: age; sex; duration of HD; previous histories of diabetes, hypertension, and cardiovascular disease (CVD); dry weight; and height. In this study, CVD was defined as heart failure, angina pectoris, myocardial infarction, and stroke. Blood samples were collected with the patient in the supine position before the initiation of the HD session on a Monday or Tuesday, and laboratory data of the month in which BIA was performed were used. Body composition was assessed using a body composition analyzer (MLT-550N, SK Medical, Japan) after a HD session. Multifrequency (2.5–350 kHz) BIA was performed using the wrist–ankle method. This approach provides information on fat mass, total body water, intracellular water (ICW), and extracellular water (ECW). Skeletal muscle mass was estimated using the following formula: skeletal muscle mass (kg) = 9.52 + 0.331 × ICW (L) + 2.77 (if male) + 0.180 × post-dialysis weight (kg) − 0.133 × age (years) [18]. Then, the FTI and SMI were calculated. BMI was calculated from dry weight and height using the following formula: dry weight (kg) / height squared (m^2^).

### Follow-up study

The study endpoint was all-cause mortality. Patients were divided into groups according to the median values of the FTI and SMI in each sex group, and thereafter, patients with higher and lower FTI/SMI values were combined into higher and lower FTI/SMI groups, respectively, as the distributions of the FTI and SMI values differ according to sex. Moreover, patients were divided into the following four groups according to the medians of both FTI and SMI values: group 1 (G1), lower FTI and lower SMI; G2, higher FTI and lower SMI; G3, lower FTI and higher SMI; and G4, higher FTI and higher SMI. The patients were followed up for as long as 5 years until March 2018.

### Statistical analysis

Normally distributed variables are expressed as mean ± standard deviation, and non-normally distributed variables are expressed as median and interquartile range. With regard to comparisons of differences among the four groups divided according to the medians of both FTI and SMI values, continuous variables were analyzed using one-way analysis of variance or the Kruskal–Wallis test, whereas categorical variables were analyzed using the chi-squared test. Multivariate regression analysis was performed to determine the factors correlated with the FTI or SMI. The Kaplan–Meier method was used to estimate survival, which was analyzed using the log-rank test. And, for multiple comparison of each group, Bonferroni correction was performed. Hazard ratios (HRs) and 95% confidence intervals (CIs) for all-cause mortality were assessed using Cox proportional hazard regression analysis. The multiple regression model included classical risk factors such as diabetes, hypertension, and history of CVD.

To assess whether the accuracy of predicting mortality would improve after the addition of the FTI and/or SMI to a baseline model with covariates that were significant at P < 0.05 in the univariate analysis, the C-index, net reclassification improvement (NRI), and integrated discrimination improvement (IDI) were calculated. The C-index was defined as the area under the ROC curves between individual predictive probabilities for mortality and the incidence of mortality, and it was compared between a model including BMI and a model including FTI and SMI [19]. NRI is a relative indicator of the number of patients for whom the predicted probabilities for mortality improve, whereas IDI represents the average improvement in predicted probabilities for mortality after the addition of variables to the baseline model [20].

All statistical analyses were performed using SPSS version 21 (IBM Corp., Armonk, NY, USA). A P-value <0.05 was considered statistically significant.

## Results

### Baseline characteristics

The baseline characteristics of the study patients are shown in Table 1. The median duration of HD was 1.9 (range, 0.7–8.2) years. The mean BMI, FTI, and SMI were 21.9 ± 3.5 kg/m^2^, 6.22 ± 3.09 kg/m^2^, and 7.64 ± 1.23 kg/m^2^, respectively. The FTI and SMI showed significant correlations with BMI (r = 0.897, P < 0.0001 and r = 0.743, P < 0.0001, respectively). And, there was a significant correlation between FTI and SMI (r = 0.488, P <0.0001). Multivariate regression analysis revealed that the FTI was independently correlated with male sex (β = −0.260, P < 0.0001) and BMI (β = 0.925, P < 0.0001) and that the SMI was independently correlated with age (β = −0.466, P < 0.0001), male sex (β = 0.392, P < 0.0001), and BMI (β = 0.610, P < 0.0001).

**Table 1:** Baseline patient characteristics.

	All patients (*N* = 162)	G1 (*N* = 55)	G2 (*N* = 26)	G3 (*N* = 26)	G4 (*N* = 55)	P-value
Age (years)	65.1 ± 12.6	71.1 ± 8.3	74.0 ± 7.8	54.2 ± 14.3	60.1 ± 11.4	<0.0001
Male (%)	68.5	65.5	73.1	73.1	67.3	0.85
Duration of HD (years)	1.9 (0.7–8.2)	1.3 (0.7–9.3)	1.6 (0.7–4.6)	6.9 (1.0–9.7)	2.3 (0.7–8.4)	0.40
Diabetes (%)	54.2	45.5	50.0	34.6	76.4	0.0006
Hypertension (%)	92.6	90.9	88.5	96.2	94.5	0.64
Smoking (%)	24.7	20.0	34.6	26.9	23.6	0.56
History of CVD (%)	46.9	43.6	53.8	34.6	52.7	0.38
BMI (kg/m^2^)	21.9 ± 3.5	18.9 ± 1.6	22.1 ± 1.4	20.8 ± 1.6	25.4 ± 2.9	<0.0001
BUN (mg/dL)	55.7 ± 14.3	53.7 ± 16.0	55.5 ± 15.1	58.7 ± 14.3	56.5 ± 12.0	0.50
Creatinine (mg/dL)	9.2 ± 2.9	8.4 ± 2.5	8.1 ± 2.7	11.1 ± 3.3	9.6 ± 2.8	0.0001
Albumin (g/dL)	3.7 ± 0.3	3.6 ± 0.4	3.6 ± 0.3	3.8 ± 0.4	3.8 ± 0.2	0.0002
Hemoglobin (g/dL)	10.9 ± 1.1	10.9 ± 1.2	10.5 ± 1.2	10.9 ± 0.8	11.0 ± 1.1	0.23
T-Cho (mg/dL)	156 ± 35	156 ± 33	144 ± 33	165 ± 36	159 ± 37	0.17
Uric acid (mg/dL)	7.2 ± 1.7	7.0 ± 2.0	6.5 ± 2.0	7.6 ± 1.3	7.5 ± 1.3	0.050
Ca (mg/dL)	8.8 ± 0.8	8.8 ± 0.9	8.6 ± 0.7	9.0 ± 1.0	8.8 ± 0.7	0.26
P (mg/dL)	5.0 ± 1.3	4.9 ± 1.3	4.7 ± 1.3	5.1 ± 1.3	5.1 ± 1.2	0.61
Glucose (mg/dL)	127 ± 40	126 ± 39	133 ± 38	107 ± 31	134 ± 44	0.031
CRP (mg/dL)	0.15 (0.05–0.35)	0.11 (0.04–0.46)	0.21 (0.06–0.34)	0.09 (0.04–0.23)	0.18 (0.10–0.40)	0.96
FTI (kg/m^2^)	6.22 ± 3.09	3.81 ± 1.65	7.25 ± 1.16	4.05 ± 0.93	9.16 ± 2.67	<0.0001
male (kg/m^2^) (N)	5.84 ± 2.92 (111)	3.38 ± 1.67 (36)	7.08 ± 1.12 (19)	3.80 ± 0.87 (19)	8.65 ± 2.28 (37)	<0.0001
female (kg/m^2^) (N)	7.03 ± 3.30 (51)	4.62 ± 1.31 (19)	7.72 ± 1.22 (7)	4.74 ± 0.76 (7)	10.20 ± 3.16 (18)	<0.0001
SMI (kg/m^2^)	7.64 ± 1.23	6.64 ± 0.74	7.02 ± 0.68	8.27 ± 0.88	8.62 ± 1.01	<0.0001
male (kg/m^2^) (N)	7.99 ± 1.13 (111)	6.99 ± 0.52 (36)	7.31 ± 0.54 (19)	8.67 ± 0.67 (19)	8.98 ± 0.86 (37)	<0.0001
female (kg/m^2^) (N)	6.86 ± 1.09 (51)	5.99 ± 0.66 (19)	6.25 ± 0.36 (7)	7.21 ± 0.27 (7)	7.87 ± 0.90 (18)	<0.0001

HD: hemodialysis, CVD: cardiovascular disease, BMI: body mass index, BUN: blood urea nitrogen, T-Cho: total cholesterol, CRP: C-reactive protein, FTI: fat tissue index, SMI: skeletal muscle mass index, G1: lower FTI and lower SMI, G2: higher FTI and lower SMI, G3: lower FTI and higher SMI, G4: higher FTI and higher SMI.

### Relationship of the FTI and/or SMI determined by BIA with mortality

**Fig 1:**
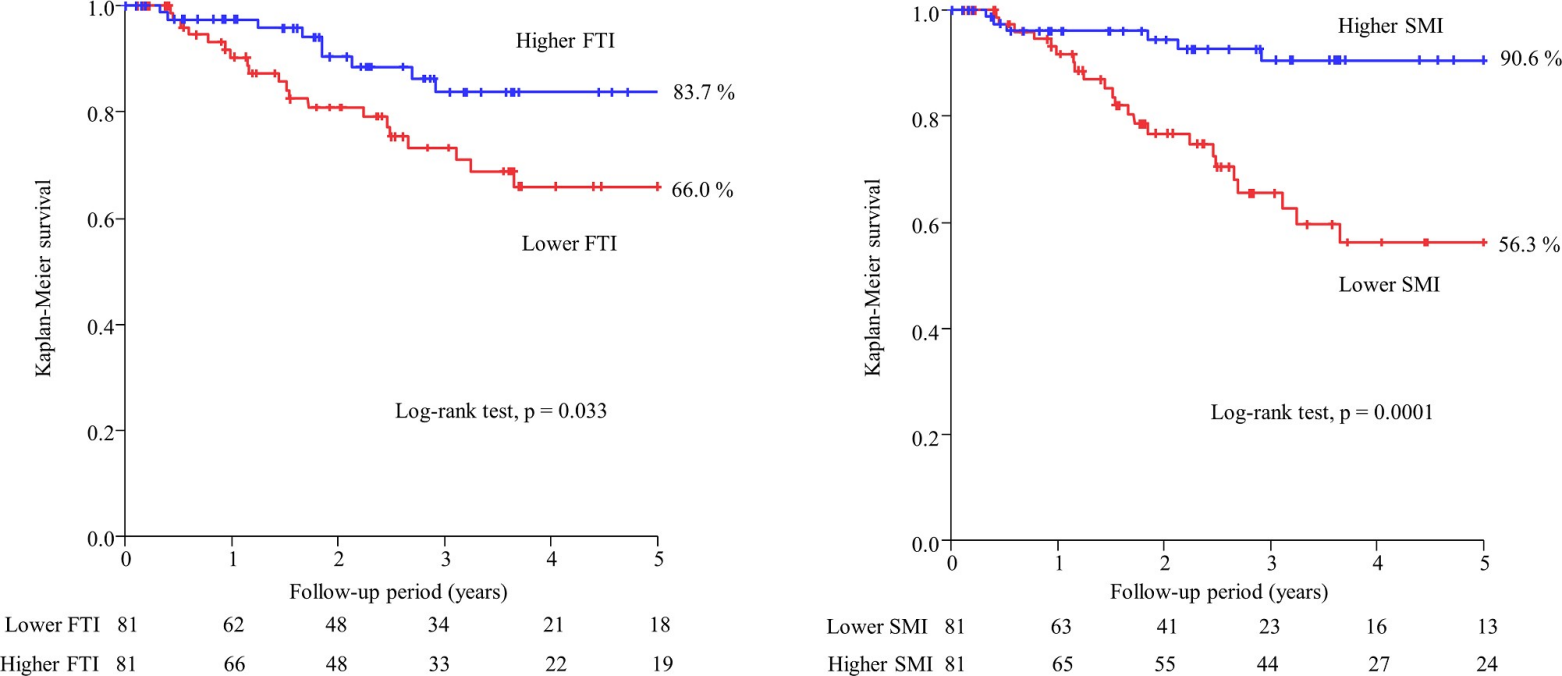
Kaplan–Meier survival curves of all-cause mortality for lower FTI vs. higher FTI and lower SMI vs. higher SMI. FTI: fat tissue index, SMI: skeletal muscle mass index.

During the follow-up period (median, 2.5 [range, 1.0–4.5] years), 29 patients died (infection, 11 [37.9%]; CVD, 6 [20.7%]; malignancy, 4 [13.8%]; and others, 8 [27.6%]). In the univariate Cox proportional hazards analysis, BMI, FTI, and SMI were significant predictors for all-cause mortality (HR 0.87, 95%CI 0.76–0.98, P = 0.022; HR 0.86, 95%CI 0.74–0.98, P = 0.021; and HR 0.60, 95%CI 0.43–0.82, P = 0.0012, respectively). The median FTI was 5.88 kg/m^2^ in males and 6.28 kg/m^2^ in females, and the median SMI was 7.93 kg/m^2^ in males and 6.86 kg/m^2^ in females. After 5 years of follow-up, the Kaplan–Meier survival rates were 66.0% and 83.7% in the lower FTI and higher FTI groups (P = 0.033) and were 56.3% and 90.6% in the lower SMI and higher SMI groups (P = 0.0001), respectively (Fig 1). In the multivariate Cox proportional hazards analysis after adjusting for age, sex, albumin, diabetes, hypertension, and history of CVD, the adjusted HR values were 0.40 (95%CI 0.17–0.90, P = 0.027) for higher FTI and 0.28 (95%CI 0.09–0.76, P = 0.011) for higher SMI (Table 2).

**Table 2:** Cox proportional hazards analysis of the risk of all-cause mortality in patients undergoing hemodialysis.

	Univariate		Multivariate	
	HR (95% CI)	P-value	HR (95% CI)	P-value
Higher FTI	0.44 (0.19−0.93)	0.031	0.40 (0.17–0.90)	0.027
Higher SMI	0.21 (0.08−0.47)	0.0001	0.28 (0.09–0.76)	0.011
Cross-classified with the medians of FTI and SMI (vs. G1)		0.0002		0.011
G2	0.41 (0.12–1.08)	0.074	0.34 (0.10–0.95)	0.040
G3	0.08 (0.01–0.37)	0.0002	0.13 (0.01–0.69)	0.013
G4	0.21 (0.07–0.53)	0.0006	0.25 (0.07–0.72)	0.0092

FTI: fat tissue index, SMI: skeletal muscle mass index, G1: lower FTI and lower SMI, G2: higher FTI and lower SMI, G3: lower FTI and higher SMI, G4: higher FTI and higher SMI. The multivariate model included age, sex, albumin, diabetes, hypertension, and history of CVD.

**Fig 2:**
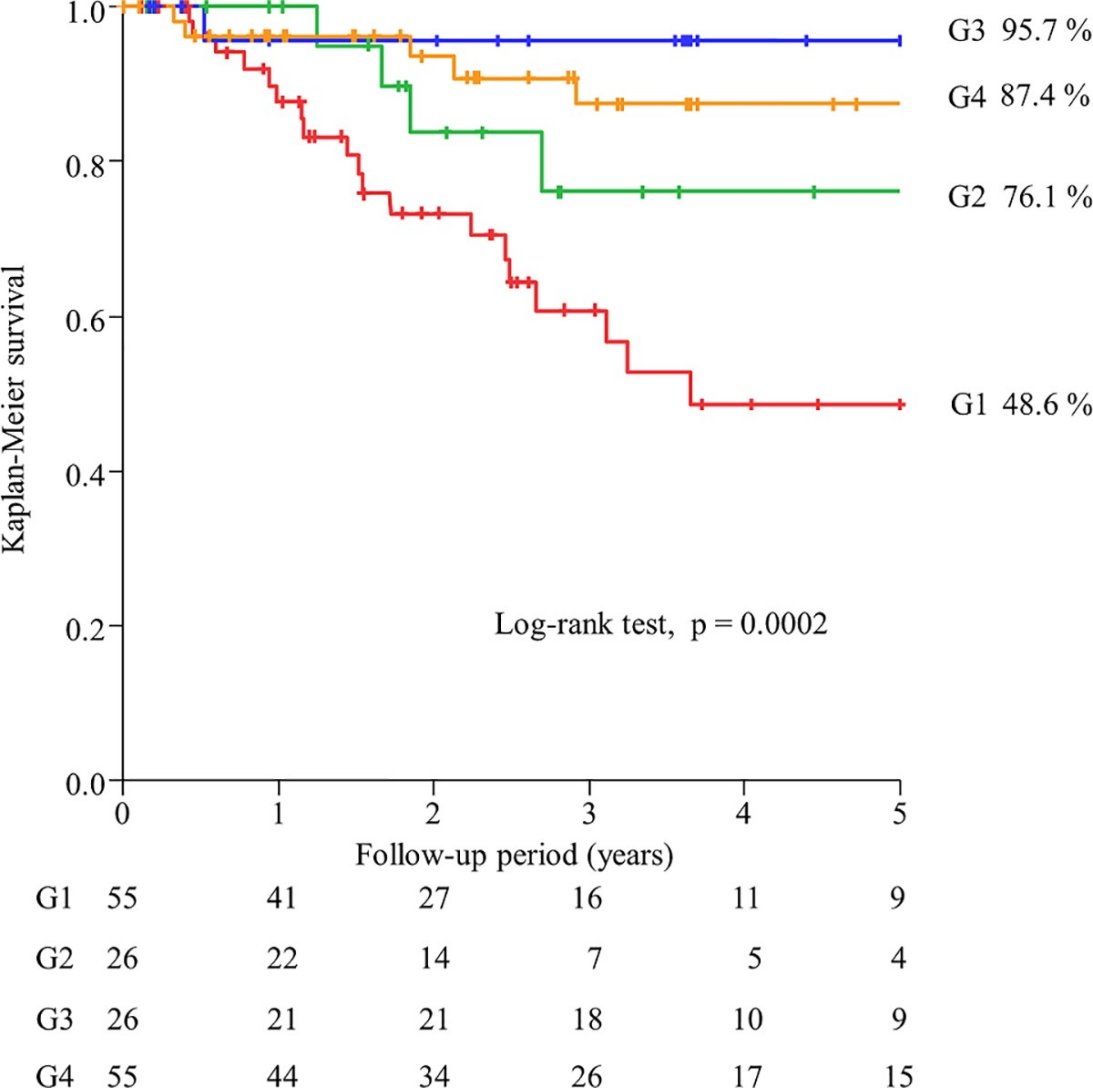
Kaplan–Meier survival curves of all-cause mortality among the four groups divided according to the medians of both FTI and SMI values. FTI: fat tissue index, SMI: skeletal muscle mass index, G1: lower FTI and lower SMI, G2: higher FTI and lower SMI, G3: lower FTI and higher SMI, G4: higher FTI and higher SM.

In G1, G2, G3, and G4, the 5-year survival rates were 48.6%, 76.1%, 95.7%, and 87.4%, respectively (P = 0.0002) (Fig 2). The survival rates of G3 and G4 were significantly higher than that of G1 (p = 0.0078 and p = 0.001, respectively). However, there was no significant differences in the survival rate between G3 and G4 (p = 0.35, even without Bonferroni correction). The adjusted HR values were 0.34 (95%CI 0.10–0.95, P = 0.040) for G2 vs. G1, 0.13 (95%CI 0.01–0.69, P = 0.013) for G3 vs. G1, and 0.25 (95%CI 0.07–0.72, P = 0.0092) for G4 vs. G1, respectively.

With regard to model discrimination, when compared with the addition of BMI to established risk factors for mortality prediction (C-index: 0.740), the addition of FTI did not improve mortality prediction (C-index: 0.740, P = 0.98; NRI: 0.1047, P = 0.70; IDI: 0.0037, P = 0.30) and the addition of SMI tended to improve mortality prediction (C-index: 0.761, P = 0.094; NRI: 0.2313, P = 0.13; IDI: 0.0149, P = 0.023). However, the addition of both FTI and SMI significantly improved mortality prediction (C-index: 0.763, P = 0.016; NRI: 0.4055, P = 0.024; IDI: 0.0158, P = 0.0070) (Table 3).

**Table 3:** Predictive values of FTI and SMI for all-cause mortality according to the C-index, net reclassification improvement (NRI), and integrated discrimination improvement (IDI).

Variable	C-Index	P-value	NRI	P-value	IDI	P-value
Established risk factors + BMI	0.740 (0.636–0.844)	Reference	Reference		Reference	
+ FTI	0.740 (0.632–0.847)	0.98	0.1047	0.70	0.0037	0.30
+ SMI	0.761 (0.664–0.859)	0.094	0.2313	0.13	0.0149	0.023
+ both FTI and SMI	0.763 (0.664–0.862)	0.016	0.4055	0.024	0.0158	0.0070

FTI: fat tissue index, SMI: skeletal muscle mass index, BMI: body mass index. Established risk factors included age, male sex, and all variables that were significant at P < 0.05 in the univariate analysis (total cholesterol and albumin).

## Discussion/Conclusion

In patients with end-stage renal disease undergoing HD, the concept of the “obesity paradox” (higher BMI is paradoxically associated with better survival) is well-known. The obesity paradox in HD patients may be partly explained by PEW, which refers to the loss of body protein and other nutritional factors leading to reduced muscle mass and fat mass. The pathophysiology of PEW is complex and can be related to a variety of factors such as malnutrition, uremia, and inflammation [1]. In the situation of inflammatory conditions or malnutrition, the storages of body protein are diverted to defend against inflammation and to repair injury. Thus, the increased body mass of overweight HD patients offers protection against or resources for responding to inflammation, infection, and subsequent CVD [4]. Moreover, given a high short-term mortality in incident hemodialysis patients, obesity–or high BMI–may be associated with better survival due to better nutritional status or larger muscle mass particularly in a short period [2].

However, BMI is not always an indicator of obesity or nutritional status, because it does not differentiate between fat mass and muscle mass [5, 6]. Thus, the limitation of BMI and the importance of measuring body composition have been recently recognized. BIA is now widely used for evaluating body composition in HD patients [9]. BIA allows not only the evaluation of the hydration status but also the differentiation of the distribution of body components, such as fat mass and muscle mass [8,9]. However, different results have been reported for the association between body composition and survival.

In the present study, a higher FTI was independently associated with a reduced risk for all-cause mortality when compared with a lower FTI. Kalantar-Zadeh et al. reported that a low body fat percentage and fat loss over time, as assessed by near-infrared interactance on the upper arm, were independently associated with high mortality in HD patients [13]. Caetano et al. reported that a lower FTI, as determined by BIA, was independently associated with a low survival rate [14]. Although high body fat is a risk factor for CVD and mortality in the general population [21,22], excess fat mass appears to be protective in HD patients [13,14]. However, it is not clear why fat possibly plays a protective role in HD patients. Fat mass is divided into visceral fat and subcutaneous fat. Visceral fat produces higher levels of inflammatory cytokines when compared with subcutaneous adipose tissue [23], and it is associated with insulin resistance and markers of oxidative stress and inflammation [24,25], which may predict the development of PEW [26,27]. On the other hand, subcutaneous fat may reflect the overall nutritional status as energy storage and may have beneficial metabolic effects, such as protection against insulin resistance [28], as observed in the general population. Additionally, it may be protective against wasting and catabolism in the setting of end-stage renal disease, particularly when intercurrent illnesses occur [29]. We have recently investigated the association between computed-tomography-measured abdominal fat levels including visceral fat and subcutaneous fat and all-cause mortality in patients undergoing HD [7]. Although the effect of subcutaneous fat on all-cause mortality is controversial in the general population, our study showed that higher subcutaneous fat, regardless of visceral fat, was independently associated with a reduced risk for all-cause mortality in HD patients [7]. In most HD patients, a high BMI likely reflects a high body fat percentage rather than muscle mass, and the fat is more likely to be non-visceral fat [30]. Thus, the positive effect of subcutaneous fat might overwhelm the negative effect of visceral fat. Therefore, a higher FTI, which reflects whole-body fat mass, may be protective with regard to survival in HD patients.

Our study also found that a higher SMI was independently associated with a reduced risk for mortality when compared with a lower SMI. Sarcopenia, which is defined as the presence of decreased muscle mass and function, is a well-known complication of PEW in HD patients [1]. However, different controversial results for the association between muscle mass and mortality have been reported. Rosenberger et al. reported that malnutrition, diagnosed as a lean tissue index (LTI: lean tissue mass adjusted by height squared; surrogate marker of muscle and determined by BIA) less than 10% of the normal value, was an independent predictor of mortality [31]. Kim et al. reported that sarcopenia, defined as both a low LTI determined by BIA (LTI of ≥2 standard deviations [SDs] below the normal sex-specific mean for young people) and low handgrip strength, was independently associated with mortality in the HD population [15]. On the other hand, Kittiskulnam et al. reported that only functional limitations, such as slow gait speed and weak handgrip strength, but not sarcopenia or low muscle mass, were predictors of mortality [16]. In their study, sarcopenia was defined as low muscle mass determined by BIA in a pre-dialysis setting (muscle mass of ≥2 SDs below the sex -specific mean for healthy young adults [18–49 years of age]) combined with weakness or slowness. There are several reasons why low muscle mass may be associated with poor survival. Low muscle mass may reflect poor nutritional status and some level of inflammation [32, 33]. It has been reported that HD patients with low muscle mass may have high levels of uremic toxins [34]. Although we could not measure gait speed or handgrip strength owing to the retrospective nature of this study, a higher SMI was significantly associated with a reduced risk of all-cause mortality in this study. Our results are similar to those of the studies by Rosenberger et al. [31] and Kim et al. [15] but are different from those of the study by Kittiskulnam et al. [16]. The difference in results between our study and the study by Kittiskulnam et al. can be explained by several factors. First, patient backgrounds differed between the studies, and the mean patient age was greater in our study (65.1 vs. 56.7 years). Second, the timing of body composition measurement differed between the studies. Body composition was measured after dialysis in our study but was measured before dialysis in the study by Kittiskulnam et al. Extracellular water is often increased in the pre-dialysis setting, and the hydration status can overestimate muscle mass [35]. Third, in the study by Kittiskulnam et al., the thresholds of muscle mass for the diagnosis of sarcopenia were determined in healthy young populations, and this might be inappropriate in HD patients.

Few studies have evaluated the association between the combination of fat mass and muscle mass and all-cause mortality, and it remains unclear whether body composition variables, such as fat mass and muscle mass, can improve mortality prediction when compared with BMI. Noori et al. reported that survival was better in individuals with a higher triceps skin-fold (TSF) thickness (an anthropometric surrogate marker of fat mass), a higher mid-arm muscle circumference (MAMC; a marker of muscle mass), and both a higher TSF and higher MAMC than in those with a lower TSF and lower MAMC [17]. On the other hand, Marcelli et al. reported that a LTI and FTI within the 10^th^ to 90^th^ percentile of an age and sex-matched healthy population were associated with the best survival, whereas a low FTI, low LTI, and especially the combination of both were associated with high mortality [10]. In the present study, higher FTI and/or higher SMI values were independently associated with reduced risks of all-cause mortality when compared with both lower FTI and lower SMI values. However, the inclusion of both FTI and SMI, but not FTI or SMI alone, significantly improved the predictive accuracy for all-cause mortality when compared with the inclusion of BMI. Therefore, these body composition indicators should be assessed simultaneously. The FTI and SMI estimated by body composition measurements may be better surrogate markers of PEW when compared with BMI, and they can explain the “obesity paradox” beyond BMI.

The present study had several limitations. First, this retrospective single-center study enrolled a relatively small number of patients. And, the number of event was very small, therefore, we could not discuss on causes of death. Second, we did not measure handgrip strength, which is important for the diagnosis of sarcopenia. Third, the duration of HD at the time of enrollment varied. Further large-scale studies are needed to validate our results.

In conclusion, higher FTI and/or higher SMI values are independently associated with reduced risks of all-cause mortality in HD patients. Moreover, the combination of the FTI and SMI can be used to stratify the risk of mortality and may more accurately predict all-cause mortality when compared with BMI. Therefore, these body composition indicators should be evaluated simultaneously in this population.

## Supporting information

S1 Data (XLSX)
